# Epidemiology of major paediatric trauma in a European Country – trends of a decade

**DOI:** 10.1186/s12887-023-03956-9

**Published:** 2023-04-25

**Authors:** Mafalda Castelão, Graça Lopes, Marisa Vieira

**Affiliations:** 1 Paediatrics Service, Department of Paediatrics, Hospital de Santa Maria, Centro Hospitalar Universitário Lisboa Norte, Av Egas Moniz, Lisbon, 1649-028 Portugal; 2 Orthopaedics Service, Hospital de Santa Maria, Centro Hospitalar Universitário Lisboa Norte, Lisbon, Portugal; 3 Paediatric Intensive Care Unit, Department of Paediatrics, Hospital de Santa Maria, Centro Hospitalar Universitário Lisboa Norte, Lisbon, Portugal

**Keywords:** Epidemiology, Paediatric, Major trauma, Public health, Prevention

## Abstract

**Objectives:**

This study investigates causes, characteristics and temporal trends of paediatric major trauma over a 10-year period and assesses potential preventive areas.

**Methods:**

Single-centre retrospective study of paediatric trauma patients admitted to a Paediatric Intensive Care Unit (PICU) in a tertiary university hospital in Europe with a level 1 paediatric trauma centre, from 2009 to 2019. Paediatric major trauma patients were defined as patients aged < 18 years with Injury Severity Score > 12, admitted for intensive care for more than 24 h following trauma. Demographic, social and clinical information, including place and mechanism of trauma, injury pattern, pre-hospital and in-hospital procedures, and length of stay in PICU was extracted from PICU medical records.

**Results:**

Total 358 patients included (age 11 ± 4,9 years; 67% male); 75% were involved in road traffic accidents: 30% motor vehicle collision, 25% pedestrian, 10% motorcycle and bicycle each. Falls from height injured 19% of children, 4% during sports activities. Main injuries were to head/neck (73%) and extremities (42%). The incidence of major trauma was highest in teenagers and did not show a decreasing trend during the study years. All fatalities (1,7%; n = 6) were related to head/neck injuries. Motor vehicle collisions resulted in higher need for blood transfusion (9 vs. 2 mL/kg, p = 0,006) and the highest ICU-mortality (83%; n = 5). Children in motorcycle accidents had longer ICU length-of-stay (6,4 vs. 4,2 days, p = 0,036). Pedestrians had 25% higher risk of head/neck injuries (RR 1,25; 1,07 − 1,46; p = 0,004), and higher incidence of severe brain injury (46% vs. 34%, p = 0,042). Most children in motor-vehicle/bicycle accidents were not using restraints/protective devices (45%) or were using them inappropriately (13%).

**Conclusions:**

Over the last decade, the absolute numbers of paediatric major trauma did not decrease. Road traffic accidents remain the leading cause of injury and death. Teenagers are at highest risk for severe trauma. Appropriate use of child restraints and protective equipment remain key for prevention.

## Background

Trauma is a major public health concern worldwide and a leading cause of death and disability in children and adolescents across all age groups, accounting for up to 35% of all childhood deaths in the developed countries [1–6]. Paediatric major trauma patients have been shown to have marked quality of life deficits at 24 months after injury [7], which can seriously interfere with the children’s development potential. The burden of trauma ranges from physiological [8] to economic [9] causes, including both the initial hospitalization and school-learning missing time, and ongoing financial and social burden due to lifelong disability and future work losses [10], resulting in a significant impact in the health and well-being of patients and families.

Despite improvements in trauma care, population studies in developed countries showed no reduction in the incidence of paediatric major trauma over the last decade [11–14]. This stationary trend calls for more action towards understanding the mechanisms and outcomes of paediatric injury for building further effective prevention strategies [15]. To date there is a lack of published reports of children and adolescents admitted to paediatric intensive care units (PICU) due to major trauma in European countries, exploring the trend of injury over the last decade. The aim of this study was to investigate causes, characteristics and temporal trends of paediatric major trauma over a 10-year period. This could allow us to suggest areas for prevention improvement.

## Methods

### Study design

A retrospective review of paediatric major trauma was conducted using data from electronic medical records, from 1 to 2009 to 1 January 2019. Data was collected from the admission notes, discharge notes, and medical daily notes, available in the Department’s databases, with permission from the dataset owner, and was kept anonymized.

### Setting

This study was conducted at the PICU of Hospital de Santa Maria, a tertiary university hospital in Lisbon, Portugal. This university medical centre includes a level 1 paediatric trauma centre, with a 24-hour in-house trauma response team, including a senior paediatric intensivist, a senior paediatric surgeon and senior neurosurgeons and orthopaedic surgeons. The referral area covered by our centre includes the Lisbon Metropolitan and the South areas of Portugal (population 4.1 million). An ambulance service provides road and air (helicopter) transport of critical patients.

### Study population

Paediatric major trauma patients were identified from the PICU database. We enrolled all consecutive patients admitted to PICU for more than 24 h following trauma during the 10-year study period, who were aged < 18 years and had an Injury Severity Score > 12 as determined by the Abbreviated Injury Scale (2005 version 2008 update). Demographic, social and clinical information, including age, sex, mode of arrival to hospital, place and mechanism of trauma, injury location, pre-hospital and in-hospital procedures, and length of stay in PICU was extracted from the PICU records.

All methods were performed in accordance with the principles stated in the Declaration of Helsinki. All experimental protocols were approved by the local Institutional Review Board – Comissão de Ética do Centro Académico de Medicina de Lisboa CAML, and written inform consent was waived.

### Data analysis

Continuous variables were expressed as medians and interquartile range (IQR) or as means and standard deviation (SD). Categorical variables were expressed as counts and percentages. Road traffic accidents were classified into motor vehicle collision, motorcycle, bicycle, and pedestrian. Comparisons of variables between groups were performed using the χ2 test and the Mann-Whitney U test, or the Student’s t-test, as appropriate. Statistical significance was defined as a two-sided p value < 0.05 in all statistical analyses. Data analysis was performed using Stata® (V.16.0, StataCorp, College Station, TX).

## Results

**Fig. 1:**
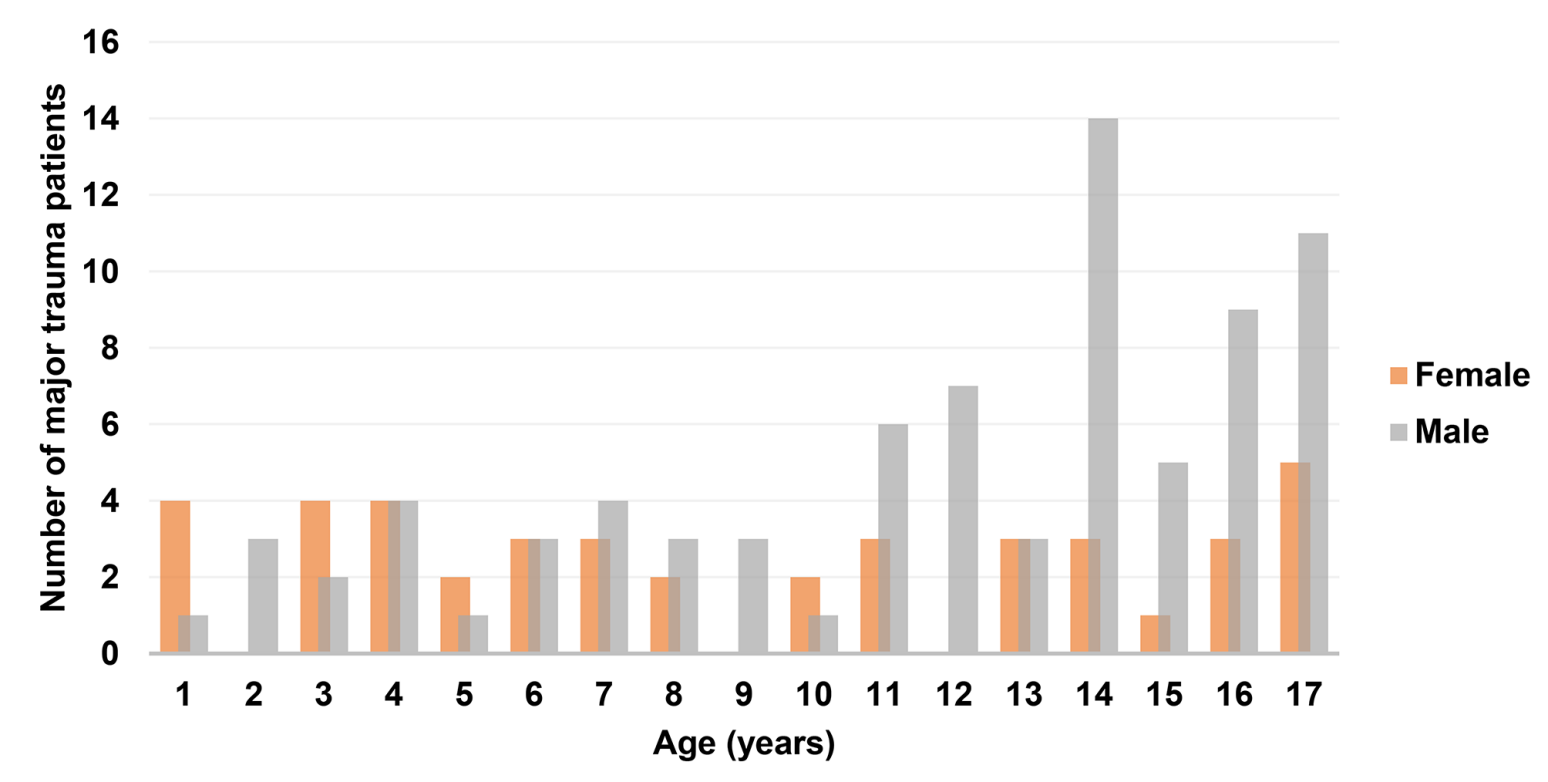
**Age distribution of all paediatric (0–18 years) major trauma patients admitted to a PICU on a level 1 trauma centre in Europe, by gender, 2009–2019 (n = 358 patients; male = 240; female = 118)**. PICU – Paediatric Intensive Care Unit

Over the 10-year study period, there were 358 paediatric major trauma patients admitted to our PICU. The demographic and clinical characteristics of the study population are reported in Table 1. Most patients were male (67%), with mean age 11 ± 4,9 years and an increased prevalence of males in teenagers (Fig. 1). Road traffic accidents were the leading mechanisms of injury (75%), of which 30% were motor vehicle collisions, 25% pedestrian, 10% motorcycle and 10% bicycle. Falls from height accounted for 19% of all paediatric major trauma cases, 4% during sport-related events. Most injuries occurred on a road, street or highway (82%), while a further 13% occurred at home and 6% in a recreation place – 3% at school, 2% at a sports area, and 1% on farms. The majority of patients (59%) were transported to our hospital directly from the trauma site, and the remaining 41% were transferred from a regional hospital after primary stabilization. Transport to the trauma centre was performed by an ambulance in 79% of cases and by helicopter in 21%.

**Table 1:** Demographic and injury event details for all paediatric (0–18 years) major trauma patients admitted to a PICU on a level 1 trauma centre in Europe, 2009–2019 (n = 358 patients)

	Total major trauma patients (n = 358)
Male, n (%)	240 (67,0)
Cause of injury*, n (%)	
Motor vehicle collision	53 (29,3)
Pedestrian	44 (24,3)
Falls	34 (18,8)
Motorcycle	18 (9,9)
Bicycle	18 (9,9)
Penetrating trauma	7 (3,9)
Suicide attempts	4 (2,2)
Work machinery	2 (1,1)
Animal attacks	1 (0,6)
Place of injury^†^, n (%)	
Road, street or highway	145 (81,5)
Home	23 (12,9)
School	5 (2,8)
Other	5 (2,8)

Missing data: *n = 181; ^†^n = 178. PICU – Paediatric Intensive Care Unit

### Patterns and trends of injury

For paediatric major trauma patients, head and neck (73%), extremities (42%) and abdominal (32%) were the most prevalent injury groups (Table 2). Most victims had multi-trauma injuries (88%) with 57% presenting with three or more injured body sites. Among the most frequent mechanisms of trauma, children involved in motor vehicle collisions sustained more head/neck and extremities injuries (77% each), those involved in pedestrian accidents suffered more head/neck and abdominal lesions (80% and 64%, respectively), while children injured by falls experienced more frequently extremities injuries (82%) followed by head/neck (59%). Children in traffic accidents in pedestrian category had 25% higher risk of suffering head and neck injuries (RR 1,25; 95% CI 1,07 − 1,46; p = 0,004) and had a higher incidence of severe brain injury (46% vs. 34%, p = 0,042), when compared to all other mechanisms of trauma. Most children in motor vehicle collision or bicycle accidents were either not using any restraints or protective devices (45%) or were using them inappropriately (13%).

**Table 2:** Patterns of injury for all paediatric (0–18 years) major trauma patients admitted to a PICU on a level 1 trauma centre in Europe, by mechanism of trauma, 2009–2019 (n = 351 patients)

	Total major trauma patients (n = 351)	Main mechanisms of trauma (n = 131)		
		Motor vehicle collision (n = 53)	Pedestrian (n = 44)	Falls (n = 34)
**Injury sites, n (%)**				
Head and neck	255 (72,6)	41 (77,4)	35 (79,5)	20 (58,8)
Extremities	148 (42,2)	41 (77,4)	21 (52,3)	28 (82,4)
Abdominal	111 (31,6)	32 (60,4)	28 (63,6)	16 (47,1)
Chest	96 (27,4)	30 (56,6)	19 (43,2)	12 (35,3)
Facial	56 (16,0)	12 (22,6)	7 (15,9)	14 (41,2)
External and other	19 (5,4)	3 (5,7)	16 (36,4)	2 (5,9)
**Number of injured body regions per patient, n (%)**				
One body region	42 (12,0)	4 (7,5)	5 (11,4)	4 (11,8)
Two body regions	110 (31,3)	10 (18,9)	11 (25)	18 (52,9)
Three or more body regions	199 (56,7)	39 (73,6)	28 (63,6)	12 (35,3)

PICU – Paediatric Intensive Care Unit

**Fig. 2:**
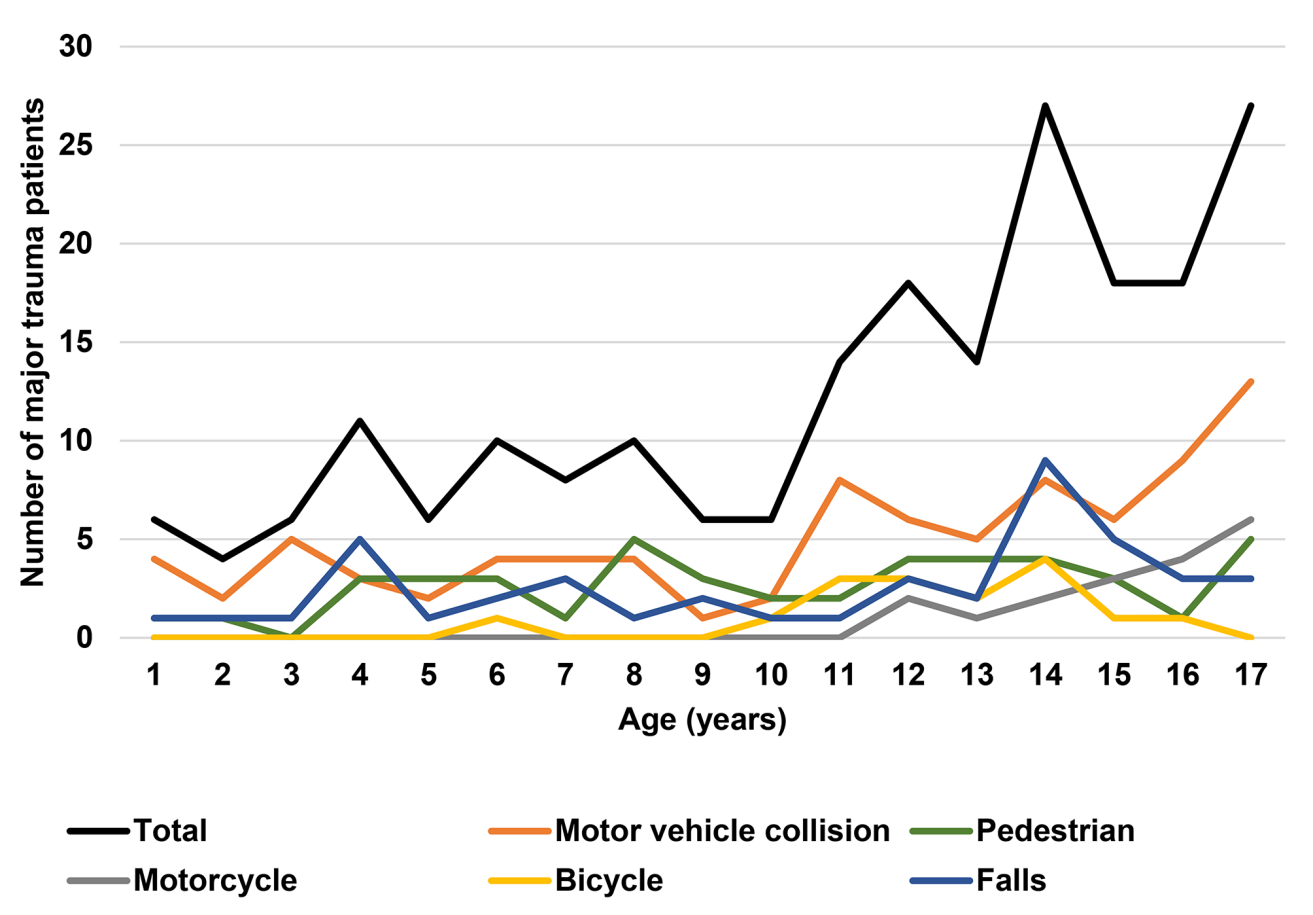
**Distribution of total number of paediatric (0–18 years) major trauma patients admitted to a PICU on a level 1 trauma centre in Europe and causes of injury, by age, 2009–2019 (n = 181 patients)**. PICU – Paediatric Intensive Care Unit

The incidence of major trauma was highest in the teenage years, with motor vehicle collisions (39%), falls (21%) and motorcycle accidents (15%) peaking as causes of injury in these age groups (Fig. 2). Two-thirds of trauma afflicting infants (aged 0–1 year) occurred in motor vehicle collisions; in pre-school children (aged 1–5), motor vehicle collisions (44%) and falls (30%) were the leading mechanisms of trauma; and in children aged 6 to 11 years old, the most frequent injuries were related to motor vehicle collisions (43%), accidents as pedestrians (30%) and falls (19%).

**Fig. 3:**
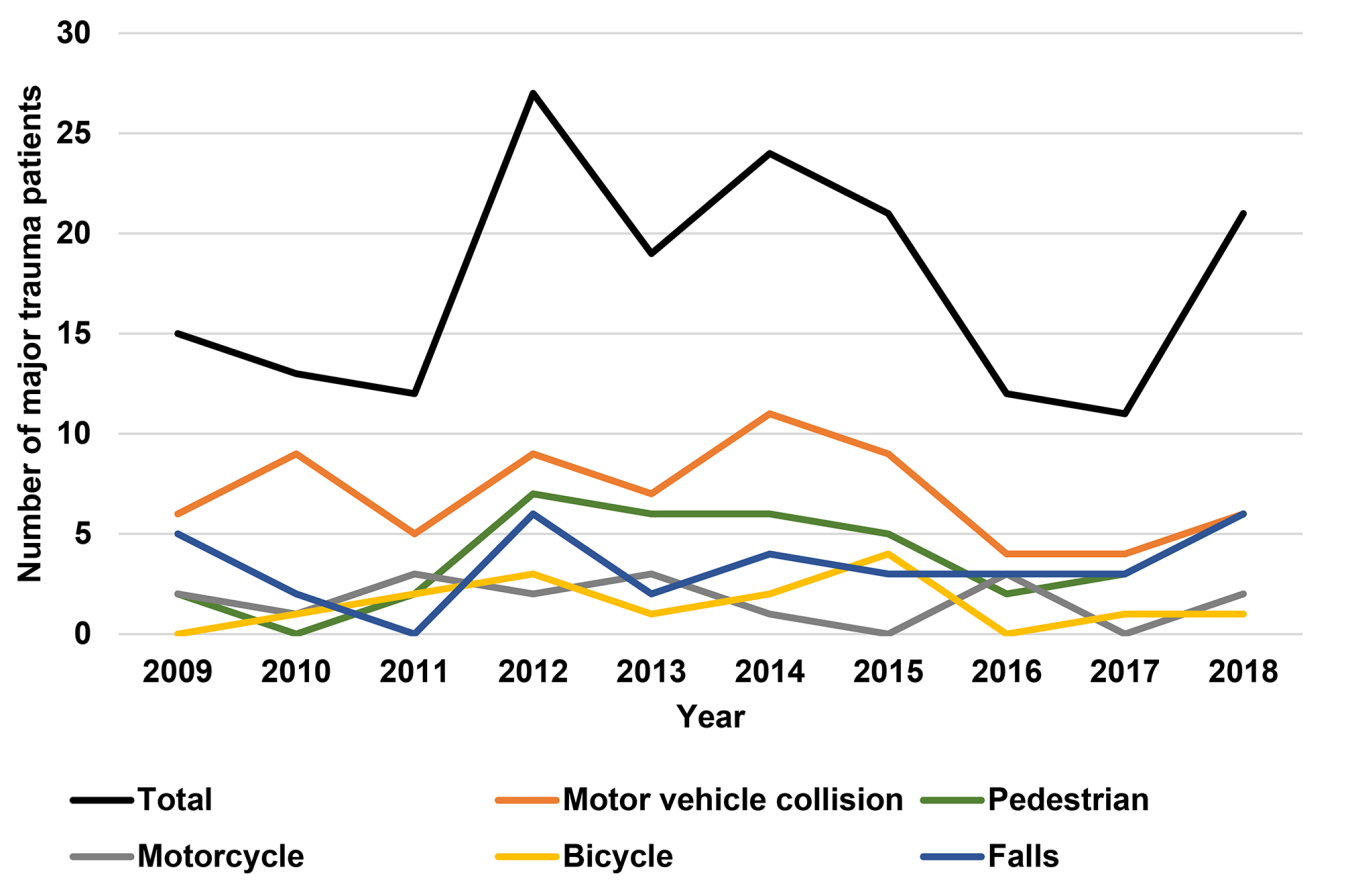
**Trend of total number of paediatric (0–18 years) major trauma patients admitted to a PICU on a level 1 trauma centre in Europe and causes of injury, by year, 2009–2019 (n = 181 patients)**. PICU – Paediatric Intensive Care Unit

The absolute number of paediatric major trauma fluctuated over the study period but did not show a decreasing trend during the decade of 2009–2019 (Fig. 3).

### Mortality and in-hospital outcomes

The overall mortality rate in PICU was 1,7% (n = 6). 67% were male, with ages between 3 and 11 years old. All fatalities were related to head and neck injuries and were caused by traffic accidents. The cause of death was severe brain injury in 5 children, and cervical spine injury in 1 child. All deaths occurred in the first 24 h post PICU admission.

The mean PICU length-of-stay was 4,7 ± 0,4 days. Motor vehicle collisions had the highest ICU-mortality (n = 5, 83%) and higher need for blood transfusion (9 vs. 2 mL/kg, p = 0,006). Motorcycle accident was the mechanism of injury requiring longer PICU admission (6,4 vs. 4,2 days, p = 0,036).

## Discussion

This study provides a description of major trauma cases admitted to a level 3 paediatric intensive care unit in a European capital, between 2009 and 2019. Over this 10-year period, there was not a consistent trend of decline in the number of paediatric major trauma. Although the number and specific causes of injury fluctuated over time, no mechanism of trauma showed a downward tendency over the last decade. Similar findings were reported by a population-based study investigating temporal trends in paediatric major trauma in Australia over a comparable period [13]. The leading causes of injury also seem to sustain over time [16], highlighting serious road safety concerns. In our study, traffic accidents accounted for three-quarters of events, and motor vehicle collisions were the leading cause of trauma throughout the decade and across all ages. Aligned with that fact, most injuries occurred on a road or street. Moreover, the surprisingly low use of safety devices in major motor vehicle collisions and bicycle events reflect the urgent need for strengthening of parental education programmes, supported by reinforcement of public and governmental measures, in order to increase compliance among population.

The incidence of major trauma was highest in teenagers and in males, and the proportion of events resulting from motorcycle and bicycle events also increased in the adolescence years. On the other hand, falls from height peaked in pre-school children and in adolescents, and injuries as pedestrians were more frequent in children aged 6 to 11 years old. These results are consistent with international studies reporting differences in causes of injury between gender and age groups [12, 16–20]. The age-related differences reflect specific risks associated to predictable stages of physical and social development in each age group, which could potentially be minimised through anticipatory education strategies and social supervising models.

Our study reports an overall PICU mortality of 1,7% for major paediatric trauma. This is within the range of previous studies that demonstrate mortality rates that vary from 1,2% to 12,8% in the USA, Canada, Australia, Japan and Europe [13, 21–26]. However, the inclusion criteria vary significantly between these studies, some including severe non-traumatic injuries such as poisoning and drowning, or patients not admitted to PICU, and therefore the results are not suitable for direct comparison. Our study identifies yet another vulnerable child group of pedestrians who have the greatest risk for head and neck trauma and severe brain injury, which was the major cause of death in all patients and represented an early mortality.

## Conclusion

Our results profiled the characteristics and trend of major paediatric trauma over the last decade. The public health significance of road traffic injuries was brought to evidence by this study, making it clear that traffic trauma prevention and safety promotion measures are still urgently required. While high-income countries have been able to substantially reduce their childhood deaths from other causes over the past decades, such as infectious diseases [16], child and adolescent injury clearly remains an emerging problem in our societies. All paediatric major trauma events reported in our study are preventable. Education and behaviour change programmes for children and parents should be incorporated across the spectrum of legislation and environment changes, but should also encompass transferring knowledge and commitment from all public health stakeholders, including physicians, patients, hospitals, pharmaceuticals and insurance companies.

## Data Availability

The datasets used and/or analysed during the current study are available from the corresponding author on reasonable request.
